# Elevated Snail Expression Mediates Tumor Progression in Areca Quid Chewing-Associated Oral Squamous Cell Carcinoma *via* Reactive Oxygen Species

**DOI:** 10.1371/journal.pone.0067985

**Published:** 2013-07-10

**Authors:** Shiuan-Shinn Lee, Chung-Hung Tsai, Cheng-Chia Yu, Yu-Chao Chang

**Affiliations:** 1 School of Public Health, Chung Shan Medical University, Taichung, Taiwan; 2 Department of Pathology, Chung Shan Medical University Hospital, Taichung, Taiwan; 3 School of Dentistry, Chung Shan Medical University, Taichung, Taiwan; 4 Department of Dentistry, Chung Shan Medical University Hospital, Taichung, Taiwan; The University of Hong Kong, China

## Abstract

**Background:**

Snail is an important transcription factor implicated in several tumor progression and can be induced by reactive oxygen species (ROS). Areca quid chewing is a major risk factor of oral squamous cell carcinoma (OSCC). Therefore, we hypothesize that the major areca nut alkaloid arecoline may induce Snail *via* ROS and involve in the pathogenesis of areca quid chewing-associated OSCC.

**Methodology/Principal Finding:**

Thirty-six OSCC and ten normal oral epithelium specimens were examined by immunohistochemistry and analyzed by the clinico-pathological profiles. Cytotoxicity, 2′, 7′-dichlorofluorescein diacetate assay, and western blot were used to investigate the effects of arecoline in human oral keratinocytes (HOKs) and oral epithelial cell line OECM-1 cells. In addition, antioxidants N-acetyl-L-cysteine (NAC), curcumin, and epigallocatechin-3 gallate (EGCG) were added to find the possible regulatory mechanisms. Initially, Snail expression was significantly higher in OSCC specimens (*p*<0.05). Elevated Snail expression was associated with lymph node metastasis (*p* = 0.031) and poor differentiation (*p* = 0.017). Arecoline enhanced the generation of intracellular ROS at the concentration higher than 40 µg/ml (*p*<0.05). Arecoline was also found to induced Snail expression in a dose- and time-dependent manner (p<0.05). Treatment with NAC, curcumin, and EGCG markedly inhibited arecoline induced Snail expression (p<0.05).

**Conclusion/Significance:**

Our results suggest that Snail overexpression in areca quid chewing-associated OSCC is associated with tumors differentiation and lymph node metastasis. Arecoline-upregulated Snail expression may be mediated by ROS generation. In addition, arecoline induced Snail expression was downregulated by NAC, curcumin, and EGCG.

## Introduction

Oral squamous cell carcinoma (OSCC) is the sixth most common type of cancer and remains as one of the leading causes of cancer-related death in the world [1], [2]. In Taiwan, OSCC is also the sixth most frequent cancer for both men and women [3]. The major risk factor in the development of OSCC is areca quid chewing [4]. Recently, accumulated evidence indicated that the possible etiology role of OSCC is the generation of reactive oxygen species (ROS) during areca quid chewing [5], [6].

Oxidative stress is the consequence of an imbalance between the production of ROS and the capacity of antioxidant scavenging mechanisms [7]. Previously, studies have indicated that elevated oxidative stress could contribute to carcinogenesis in many types of cancer cells [8], [9]. Moreover, studies have also demonstrated that oxidative stress is associated with the formation of OSCC [6], [10]. However, the possible pathogenic mechanisms of ROS in areca quid chewing-associated OSCCs still remain to be elucidated.

Epithelial–mesenchymal transdifferentiation (EMT), an important mechanism during early steps of tumor progression and which is a critical process involved in the transdifferentiation of polarized epithelial cells into an invasive mesenchymal phenotype, which acts as a critical mechanism mediating embryogenesis and cancer metastasis [11], [12]. Snail, a member of the Snail family of zinc finger transcription factors, is one of the master regulators that promotes EMT and mediates invasiveness as well as metastasis in many different types of malignant tumors [13], [14]. In addition, Snail is also reported to be triggered with ROS and associated with tumor progression [15], [16]. Overexpression of Snail is found in various cancers, including ovarian [17], breast [18], gastric [19], colorectal [20], and oral cancer [21], [22].

Althought the overexpression of Snail in OSCC specimens has been previously reported [21], [22], the possible mechanisms of increased Snail expression in areca quid chewing-associated OSCCs are still unclear. The present study was undertaken to investigate whether Snail expression in areca quid chewing-associated OSCCs, and further to test the effects of ROS generation and snail expression by arecoline, the major areca nut alkaloid, in human oral keratinocytes (HOKs) and oral cancer cell line OECM-1 by using DCFH-DA assay and Western blot. Moreover, glutathione (GSH) precursor N-acetyl-L-cysteine (NAC), curcumin, and epigallocatechin-3-gallaten (EGCG) were added to find the possible protective effect on arecoline-induced Snail expression.

## Materials and Methods

### Chemicals and Materials

Arecoline, NAC, curcumin, and EGCG were purchased from Sigma-Aldrich (St. Louis, MO, USA). All culture materials were purchased from GIBCO (Grand Island, NY, USA). Snail antibody (L70G2, #3895S) was purchased from Cell Signaling Technology (Cell Signaling Technology, Inc., Danvers, MA, USA). Arecoline, NAC, were directly dissolved in the culture medium. Curcumin and EGCG were first dissolved in dimethyl sulfoxide (Sigma-Aldrich, St. Louis, MO, USA) and then diluted with culture medium. The final concentration of solvent in the medium did not exceed 0.25% (v/v). At these concentrations of solvents used were not cytotoxic to HOKs and OECM-1 cells. In this study, the final concentrations of NAC, curcumin, and EGCG used were 1 mM, 20 µM, and 10 µM, respectively.

### Immunohistochemistry

This study was approved by the Institutional Review Board of Chung Shan Medical University Hospital and informed written consent to participate in the study was obtained from each individual (CSMUH No: CS11007). Oral-cancer patients were clinically staged at the time of their diagnosis according to the TNM staging system of the American Joint Committee on Cancer (AJCC). Tumor differentiation was examined by a pathologist according to the AJCC classification. We deparaffinized and rehydrated 5-µm section of formalin-fixed, paraffin-embedded specimens and stained with the monoclonal anti-Snail antibody (1∶50 dilution) using a standard avidin-biotin-peroxidase complex method as described previously [23]. Diaminobenzidine (Zymed, South San Francisco, CA, USA) was then used as the substrate for localizing the antibody binding. Negative controls included serial sections from which either the primary or secondary antibodies were excluded. The preparations were counterstained with hematoxylin, mounted with Permount (Merck, Darmstadt, Germany) and examined by light microscopy. Snail staining in the tumor cell nucleus was defined as positive staining. Pathologists scoring the immunohistochemistry were blinded to the clinical data. The interpretation was done in 5 high-power views for each slide, and 100 cells per view were counted for analysis. Processed immunohistochemically for Snail expression, sections graded as ‘low’ were represented by positive stained cells <10%; sections graded ‘high’ exhibited positive stained cells over 10% on three sections/tissue at 200×magnification as described previously [24].

### Cell Culture

Primary HOKs purchased from ScienCell Research Laboratories (San Diego, CA, USA) were cultured in a keratinocyte growth medium (ScienCell Research Laboratories Inc., San Diego, CA) as described previously [25]. Oral epithelial cell line OECM-1 was grown in RPMI 1640 medium (Gibco Laboratories, Grand Island, NY, USA) supplemented with 10% fetal calf serum (FCS) (Gibco Laboratories, Grand Island, NY, USA) and antibiotics (100 U/ml of penicillin, 100 µg/ml of streptomycin and 0.25 µg/ml of fungizone).

### Cell Viability Assay

Cell viability was analyzed by using WST-1 assay which is a colorimetric assay based on the reduction of tetrazolium salt to water soluble formazan [26]. Briefly, cells were detached with trypsin (GIBCO, Grand Island, NY, USA) and resuspended in adhesive medium at a concentration of 5×10^5^ cell/ml. Thereafter, 2×10^4^ HOKs and OECM-1 per well were seeded to 96-well plate and left overnight to attach. Serial dilutions of arecoline (0–160 µg/ml) in 100 µl volumes were added, and cells were treated for 24 h. The resulting cell suspensions were placed in 96-well plates in 100 µl of medium together with 10 µl of a working solution containing WST-1 Assay Kit (Bio-Chain Institute, Hayward, CA, USA) and incubated for 2 h at 37°C. The absorbances of the coloured product were measured using a spectrophotometer (Metertech Co., Taipei, Taiwan). The optical density values were expressed as a percentage of formazan formation.

### Measurement of Intracellular ROS Generation

The semi-quantitative 2′, 7′-dichlorfluorescein-diacetate (DCFH-DA) fluorescence technique was used to detect the intracellular level of ROS [27]. Briefly, 2×10^4^ cells were grown in 96-well plate for 24 h, and then incubated with 20 µM DCFH-DA (Sigma-Aldrich, St. Louis, MO, USA) for 30 min in the dark. DCFH-DA is a non-fluorescent dye easily to permeate into cells and then be hydrolyzed by intracellular esterase to DCFH storage in cells. At the end of DCFH-DA incubation, cells were washed with PBS. Cells were then treated with different concentrations of arecoline for 8 h. After washing, the formation of fluorescence dichlorofluoroscein (DCF), which is the oxidized product of DCFH-DA in the presence of several ROS primarily hydroperoxide, was measured by using the fluorescence microplate reader (Molecular Devices, CA, USA) at excitation/emission wavelengths of 400/505 nm. Results were expressed as the fluorescence intensity.

### Snail Expression Analysis

HOKs and OECM-1 were arrested in G_0_ by serum deprivation according to our previous experiments [28]. Nearly confluent monolayer of HOKs and OECM-1 were washed with serum-free medium and immediately thereafter exposed at the indicated incubation times to 0, 20, 40, and 80 µg/ml arecoline. Cell lysates were collected at 8 h for Western blot analysis. Cultures without FCS were used as negative control. Subsequently, arecoline and various pharmacological agents without cytotoxic concentrations were added to test the regulation effects during 8 h coincubation period.

### Western Blot Assay

Cell lysates were collected as described previously [24]. Briefly, cells were solubilized with sodium dodecyl sulfate-solubilization buffer (5 mM EDTA, 1 mM MgCl_2_, 50 mM Tris-HCl, pH 7.5 and 0.5% Trition X-100, 2 mM phenylmethylsulfonyl fluoride, and 1 mM N-ethylmaleimide) for 30 min on ice. Then, cell lysates were centrifuged at 12,000 g at 4°C, and the protein concentrations determined with Bradford reagent using bovine serum albumin as standards. Equivalent amounts of total protein per sample of cell extracts were run on a 10% sodium dodecyl sulfate-polyacrylamide gel electrophoresis and immediately transferred to nitrocellulose membranes. The membranes were blocked with phosphate-buffered saline containing 3% bovine serum albumin for 2 h, rinsed, and then incubated with primary antibodies anti-Snail (1∶1000) in phosphate-buffered saline containing 0.05% Tween 20 for 2 h. After three washes with Tween 20 for 10 min, the membranes were incubated for 1 h with biotinylated secondary antibody diluted 1∶1000 in the same buffer, washed again as described above and treated with 1∶1000 streptavidin-peroxidase solution for 30 min. After a series of washing steps, protein expression was detected by chemiluminescence using an ECL detection kit (Amersham Biosciences UK Limited, Buckinghamshire, UK), and relative photographic density was quantitated by scanning the photographic negatives on a gel documentation and analysis system AlphaImager 2000 (Alpha Innotech Corp., San Leandro, CA, USA). Each densitometric value was expressed as the mean ± SD.

### Statistical Analysis

The statistical significance of differences between Snail staining grade and various clinicopathologic features such as T-categories, N-categories, sex, staging of the patients, and cell differentiation of OSCCs were assessed by the Fisher’s exact test for independence. All assays were repeated three times to ensure reproducibility. Statistical analysis was carried out by student’s *t* test and a value of *p*<0.05 was considered statistically significant.

## Results

### Expression of Snail in OSCCs

**Figure 1:**
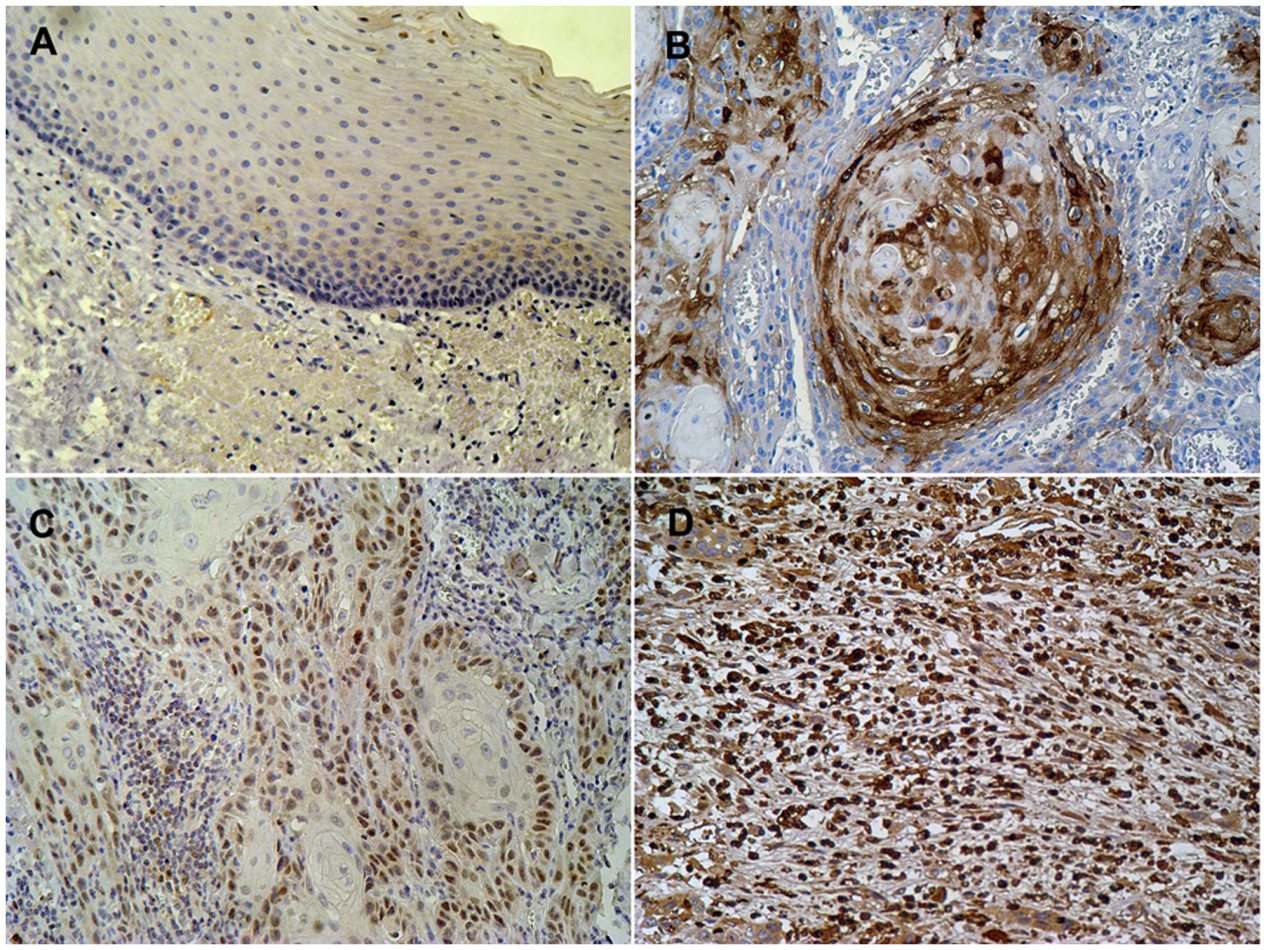
Expression patterns of Snail in OSCC specimens. (A) Very faint immunoreactivity of Snail was observed in normal epithelial specimens (200X). (B) In well-differentiated oral squamous cell carcinomas (OSCCs), low immunoreactivity expression surrounding the epithelial pearl, while centrally situated cells remained weakly positive (200X). (C) In moderately differentiated OSCCs, homogeneous and moderate staining for Snail was labeled in diffuse type at the peripheral of the nest (200X). (D) In poorly differentiated OSCCs, individual tumor cells were observed for Snail labeling and also had a stronger Snail staining (200X).

Snail staining was mainly expression in OSCC specimens and the intensity was significantly higher than normal epithelium (Fig. 1A). As shown in figure 1B, high immunoreactivity expression was shown in nuclei around cancer cell nests in the well-differentiated OSCCs, while centrally situated cells remained negative. In moderately differentiated OSCCs, intensitive staining for Snail was labeled in diffuse type at the peripheral of nest (Fig. 1C). In poorly differentiated OSCCs, a mosaic pattern, strong nuclear Snail staining were observed (Fig. 1D).

### Correlation between Snail Overexpression with Clinco-pathologic Features in OSCCs

With regard to the correlation with Snail expression and clinico-pathological parameters, table 1 shows the correlation between Snail expression level and the clinico-pathological features of these cases. Subjects were divided into two age groups, greater than or less than the median (54 years). The immunostaining of Snail in OSCC could be classified into two groups: one was low grade, 52.7% (19/36) and the other was high grade, 47.3% (17/36). No significant difference in Snail expression was observed with respect to other factors, such as age, sex, T category, and stage (*p*>0.05). The high level of Snail expression was observed in the poor differentiated groups (*p* = 0.0017). Moreover, the higher Snail expression level was significantly associated with lymph nodes metastasis (*p* = 0.031).

**Table 1:** Correlation with Snail expression and clinicopathological features.

	High expression	Low expression	*p*-value
**Age**			
>54	10	8	1.0
< = 54	9	9	
**Sex**			
Female	4	1	0.342
Male	15	16	
**T category**			
T1+2	11	11	0.742
T3+4	8	6	
**N category**			
N0	10	15	0.031
N1-2	9	2	
**Stage**			
I – II	8	10	0.505
III – IV	11	7	
**Differentiation**			
Well	4	11	0.017
Moderate or poor	15	6	

Statistical analysis was evaluated by fisher’s exact test. *p*<0.05 was considered statistically significant.

### Cell Viability Assay of HOKs and OECM-1 Stimulated with Arecoline

**Figure 2:**
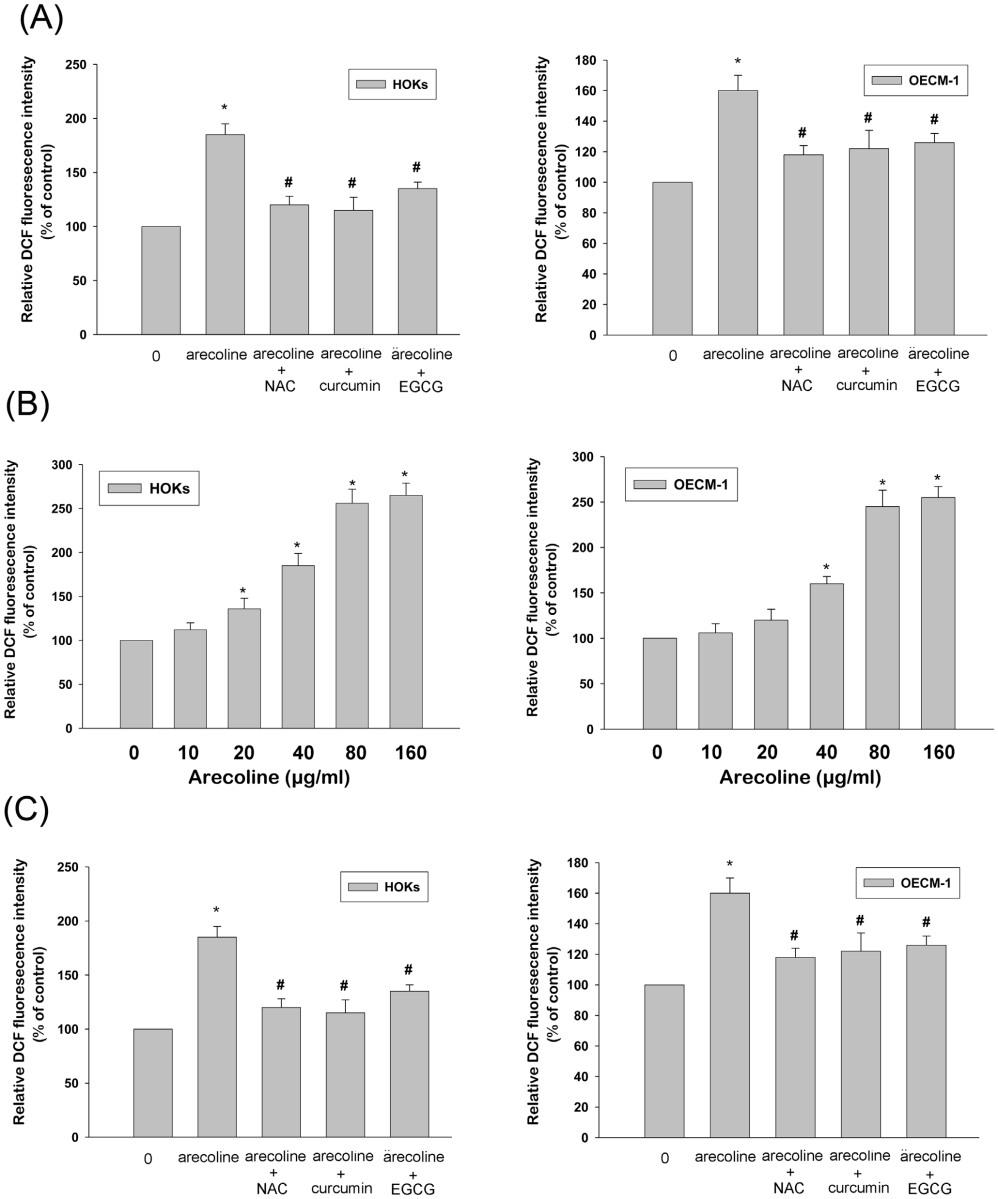
Effects of arecoline on ROS in generation in HOKs and OECM-1 cells. (A) Effects of various concentrations of arecoline on cell growth of HOKs and OECM-1. (B) Effects of arecoline on intracellular ROS generation. HOKs and OECM-1 were stimulated with the indicated concentrations of arecoline. (C) The regulatory effects of NAC, curcumin, and EGCG on arecoline-induced intracellular ROS generation in HOKs and OECM-1 cells. Cells were coincubation with pharmacological agents in the presence of 40 µg/ml arecoline. Results are expressed as percentages of absorbance relative to untreated control. Data are shown as the mean of three independent experiments ± SD. * represents significant differences from control values with *P*<0.05, # represents statistically significant between arecoline alone and arecoline with pharmacological agents; *p*<0.05.

The cytotoxicity of various concentrations (0–160 µg/ml) of arecoline on HOKs and OECM-1 for 24 h by WST-1 assay was shown in Figure 2. It was clear that the treatment of arecoline, at concentrations ranging from 0–40 µg/ml, has no cytotoxic effects to HOKs and OECM-1 during 24 h incubation period (p>0.05). However, the concentrations higher than 80 µg/ml, arecoline demonstrated cytotoxicity to HOKs and OECM-1 (p<0.05). The concentration up to 160 µg/ml, the cell viability of HOKs and OECM-1 were 64% and 67% as compared with control, respectively (Fig. 2A).

### Arecoline Iinduced Intracellular ROS Generation in HOKs and OECM-1

Effects of arecoline on intracellular ROS generation, as shown in figure 2B, the exposure of HOKs and OECM-1 to arecoline evoked intracellular ROS generation in a concentration-dependent manner (p<0.05) by DCFH-DA. The concentrations of arecoline higher than 20 µg/ml significantly upregulated the intracellular ROS generation (p<0.05) in HOKs. The levels of intracellular ROS were increased about 1.1, 1.4, 1.9, 2.6, and 2.7 fold after exposure to arecoline for 10, 20, 40, 80, and 160 µg/ml, respectively. While in OECM-1, the concentrations of arecoline higher than 40 µg/ml significantly upregulated the intracellular ROS generation (p<0.05) in OECM-1. The levels of intracellular ROS were increased about 1.1, 1.2, 1.6, 2.5, and 2.6 fold after exposure to arecoline for 10, 20, 40, 80, and 160 µg/ml, respectively (Fig. 2B). Moreover, the addition of NAC, curcumin, and EGCG significantly reduced arecoline-induced ROS generation both in HOKs and OECM-1(Fig. 2C).

### Arecoline Increased Snail Expression in a Dose- and Time-dependent Manner

**Figure 3:**
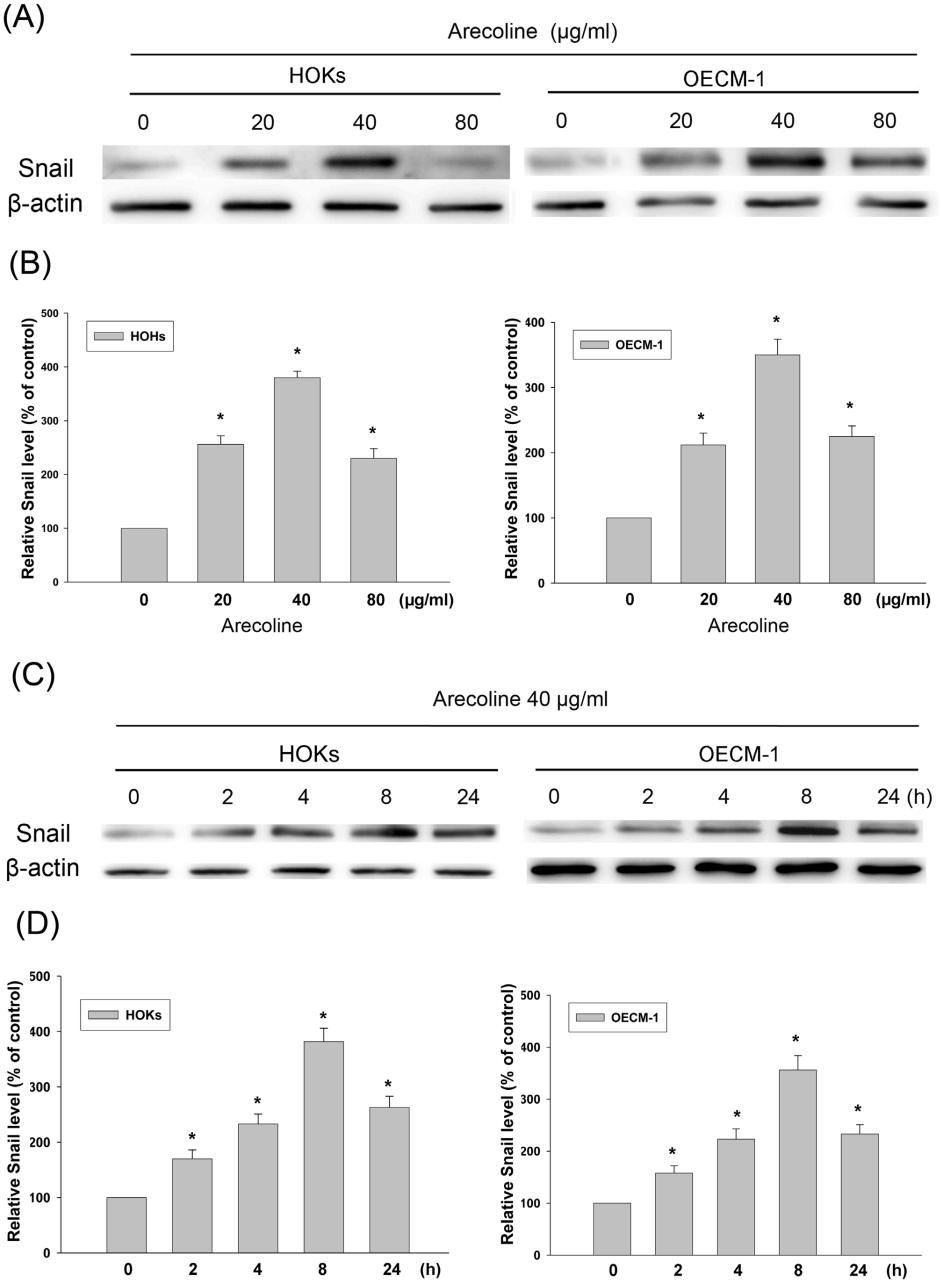
Expression of Snail in arecoline-treated HOKs and OECM-1 by Western blot. (A) Cells were exposed for 8 h in medium containing various arecoline concentrations as indicated. (B) Levels of Snail protein treatment with arecoline were measured by densitometer. (C) Kinetics of Snail expression in HOKs and OECM-1 cells exposed to 40 µg/ml arecoline for 0, 2, 4, 8, and 24 h, respectively. (D) Levels of Snail protein treated with arecoline were measured by densitometer. The relative level of Snail protein expression was normalized against β-actin signal and the control was set as 1.0. Optical density values represent the mean ± SD. * represents significant difference from control values with *p*<0.05.

Western blot was used to verify whether arecoline could affect Snail protein expression in HOKs and OECM-1. As shown in figure 3A, arecoline was found to elevate Snail expression in a dose-dependent manner (*p*<0.05) both in HOKs and OECM-1. From the AlphaImager 2000 (Fig. 3B), the amount of Snail was elevated about 2.5, 3.8, and 2.3 fold after exposure to 20, 40, and 80 µg/ml arecoline (*p*<0.05), respectively in HOKs. In OECM-1, the amount of Snail was elevated about 2.1, 3.5, and 2.3 fold after exposure to 20, 40, and 80 µg/ml arecoline (*p*<0.05), respectively. Moreover, the peak of Snail protein level induced by arecoline was 40 µg/ml. On the basis of these results, experiments described below were performed at a concentration of 40 µg/ml arecoline. Investigations of the time-dependence of Snail protein expression in 40 µg/ml arecoline-treated HOKs and OECM-1 were found to be significantly enhanced (*p*<*0.05*). The kinetics of this response showed that Snail protein was first detectable in cell lysates at 2 h post-arecoline challenge and remained elevated throughout the 24 h incubation period (Fig. 3C). From the AlphaImager 2000 (Fig. 3D), the amount of Snail protein increased about 1.7, 2.3, 3.8, and 2.6 fold after exposure to arecoline for 2, 4, 8, and 24 h (*p*<0.05), respectively in HOKs. In OECM-1, the amount of Snail protein increased about 1.6, 2.2, 3.5, and 2.3 fold after exposure to arecoline for 2, 4, 8, and 24 h (*p*<0.05), respectively.

### Antioxidants Repress Snail Expression Induced by Arecoline

**Figure 4:**
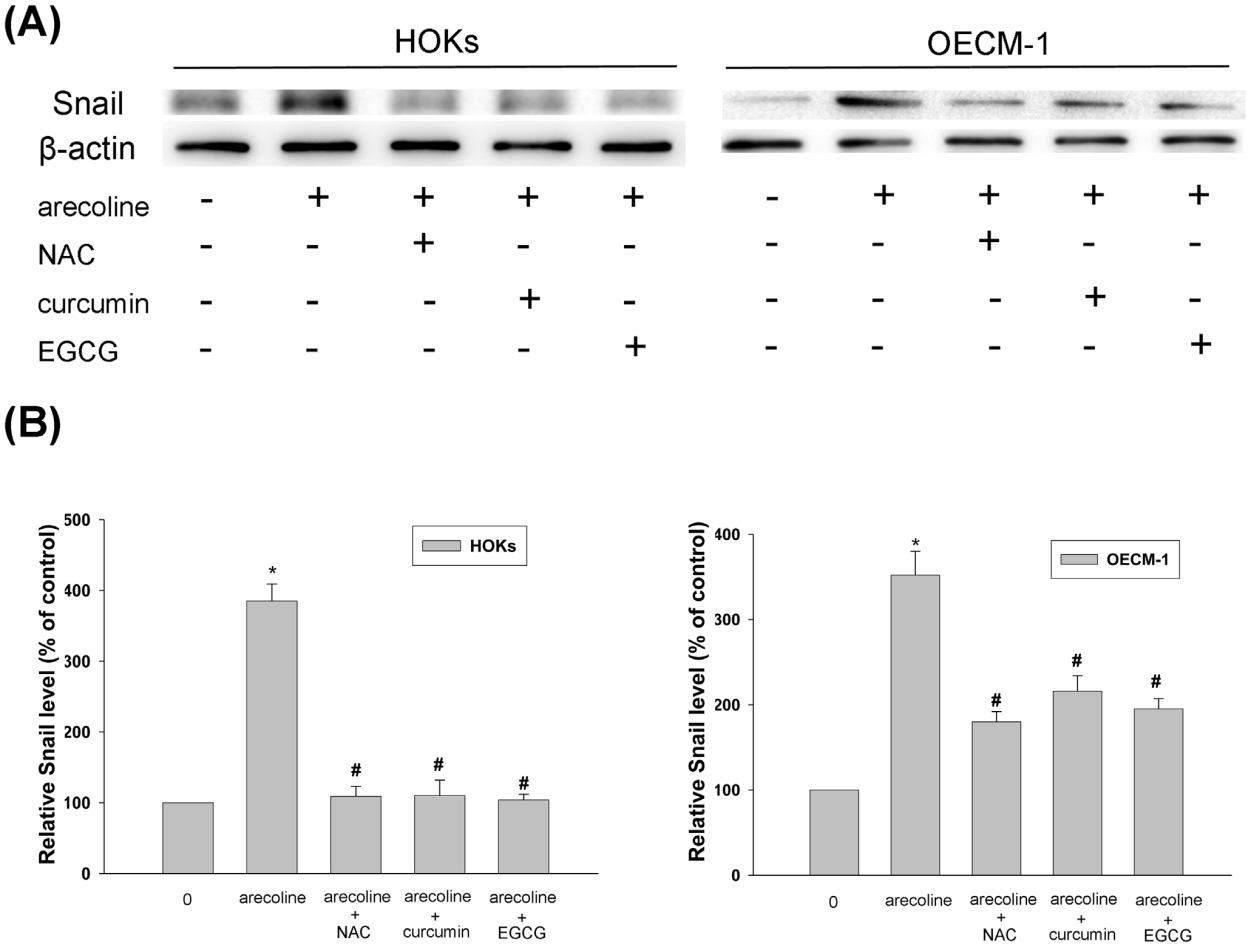
The regulatory effects of NAC, curcumin, and EGCG on arecoline-induced Snail expression in HOKs and OECM-1 cells. (A) Cells were coincubation with pharmacological agents in the presence of 40 µg/ml arecoline. β-actin was performed in order to monitor equal protein loading. (B) Quantization was achieved by densitometer as described in figure 3. * represents significant difference from control values with *p*<0.05. # represents statistically significant between arecoline alone and arecoline with pharmacological agents; *p*<0.05.

NAC, curcumin, and EGCG without cytotoxic concentrations were added to search the possible regulatory mechanisms on arecoline-induced Snail expression. These pharmacological agents were found to inhibit arecoline-induced Snail expression (*p*<0.05) (Fig. 4A). From the AlphaImager 2000 (Fig. 4B), NAC, curcumin, and EGCG were found to reduce arecoline-induced Snail expression by suppressing 2.7, 2.7, and 2.8 fold (*p*<0.05), respectively in HOKs. In OECM-1, NAC, curcumin, and EGCG were found to reduce arecoline-induced Snail expression by suppressing 1.7, 1.4, and 1.6 fold (*p*<0.05), respectively.

## Discussion

The Snail family of transtription factors is one of the key regulators of epithelial to mesenchymal transition in normal development and during tumor progression [13], [14]. Overexpression of Snail has been reported in various cancers [15]–[22], but its mechanistic roles in areca quid chewing-associated OSCCs and the contributions to carcinogenetic process have remained to be elucidated. The present study demonstrated for the first time to evaluate the expression of Snail in areca quid chewing-associated OSCCs both in vivo and ex vivo. To the best of our knowledge, Snail expression was first found to highly expressed in areca quid chewing-associated OSCCs than normal epithelium. Similar results were found in previously studies that Snail was highly expressed in OSCC specimens [21], [22].

In the present study, we also investigated the relationship between Snail expression and the clinical features of areca quid chewing-associated OSCCs. The Snail expression was significantly associated with lymph node metastasis. Our results were in agreement with Mendelsohn *et al*., [29] who reported the correlation between high Snail expression and tumor metastasis in head and neck squamous cell carcinoma. However, our results differed from those of several studies which have demonstrated that no correlation between Snail expression and metastasis in OSCCs [21], [22]. The contradictory results may be due to the different origins of specimens, different causation of OSCC, or different experimental protocols used in each laboratory.

With regard to Snail expression and histological differentiation, the enhanced expression of Snail was dramatically associated with poor histological differentiation in this study. Our results are in line with those of several studies which have reported that Snail expression was strongly correlated with poor differentiation in OSCCs [21] and head and neck squamous cell carcinoma [29]. However, our results were differed from Marcus et al., [22] who reported that Snail expression shows no correlation to differentiation in OSCCs. The contradictory results may be due to the different origins of specimens, or different experimental protocols used in each laboratory.

In the present study, we found that cell viability was in general cytostatic during exposure to 0–160 µg/ml arecoline during 24 h cultured period. Arecoline exhibited cytotoxicity at the concentrations higher than 80 µg/ml. Similar studies were reported previously that arecoline had no effects on the cell proliferation in lower doses and inhibited cell growth in higher doses in human gingival fibroblasts [30] and human buccal fibroblasts [31]. Oxidative stress was cited as an important role in promoting cytotoxicity and previous studies have demonstrated that elevated oxidative stress could contribute to carcinogenesis in many type cancer cells [8], [9]. In the present study, we found that intracellular ROS generation could be induced by arecoline in HOKs and OECM-1. The results were in agreement with previous studies that arecoline could induce ROS generation in OECM-1 and esophageal carcinoma cell line CE81T/VGH [32].

In this study, we first reported that arecoline increased Snail protein expression in HOKs and OECM-1. This suggests that one of the pathogenic mechanisms of OSCCs may be the synthesis of Snail expression by epithelial cells in response to areca quid challenge. In addition, it has been reported that intracellular ROS can induce Snail expression [16]. Taken together, Snail elevated by arecoline stimulation in HOKs and OECM-1 may be mediated by intracellular ROS.

The activation of Snail in areca quid chewing-associated OSCCs might be explained as follows. Snail is upregulated by oxidative stress. Increasing evidences have demonstrated that the generation of ROS was found during areca nut chewing [5], [6]. Consequently, we suggest that ROS generation may at least in part be responsible for the upregulation of Snail in areca quid chewing-associated OSCCs. NAC, curcumin, and EGCG are well known antioxidants. NAC is a thiol and mucolytic agent, which can act both as a precursor of reduced glutathione and as a direct ROS scavenger [33]. Curcumin, the main active component of turmeric isolated from the plant *curcuma longa L*. and which is well known as a potent scavenger of reactive oxygen and nitrogen species such as hydroxyl radicals and nitrogen dioxide radicals [34]. EGCG is the most abundant polyphenol in green tea and act as antioxidant and supress tumor promotion through reduction of ROS generation [35]. In the present study, arecoline was found to induce intracellular ROS generation in HOKs and OECM-1. The addition of NAC, curcumin, and EGCG were found to inhibit arecoline-induced Snail expression. Taken together, these results indicated that NAC, curcumin, and EGCG may play an antioxidative role in the reduction of arecoline-induced Snail expression. Dietary chemoprevention might be a promising cost-effective and safe approach in prevention ROS-induced tumor progression. Therefore, increasing dietary intake or dietary supplements of NAC, curcumin, and EGCG may have chemopreventive potential to reduce areca quid chewing-associated OSCCs.

**Figure 5:**
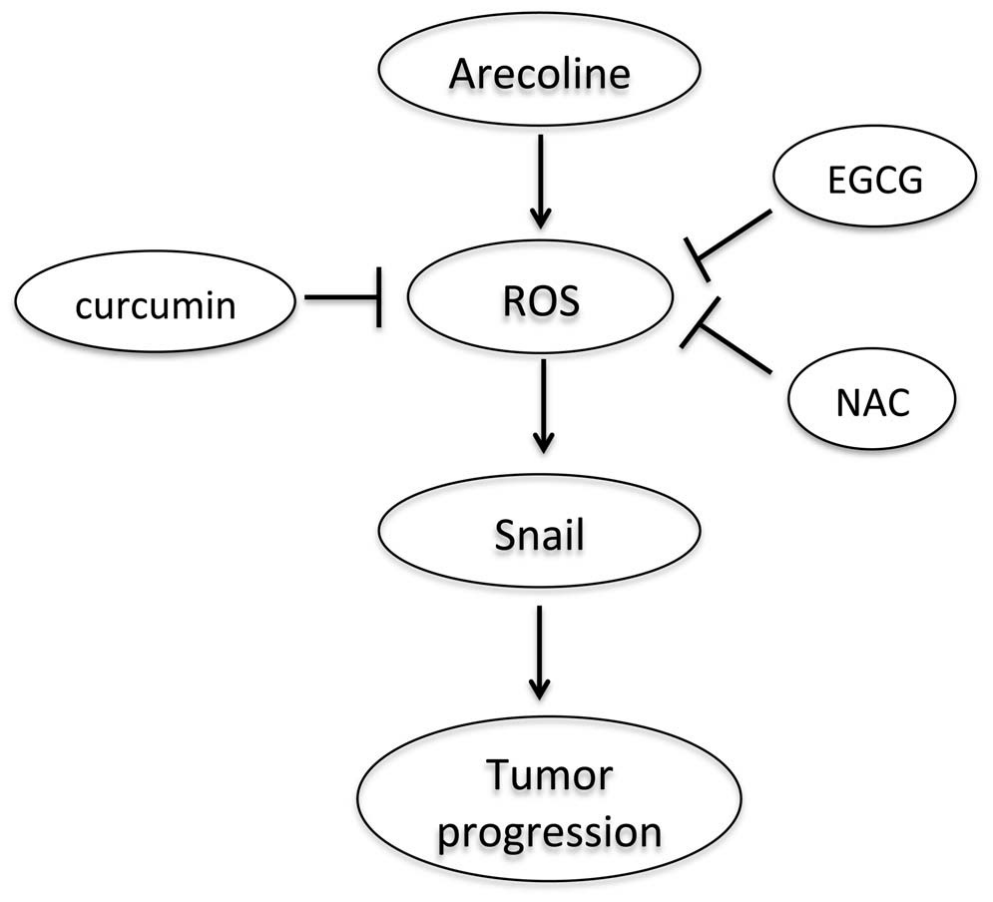
Schematic of the possible mechanisms involved in arecoline-induced Snail expression in HOKs and OECM-1 cells.

In summary, this study represents that Snail is elevated in OSCCs specimens from areca quid chewers. The elevated Snail expression was associated with lymph node metastasis. These results suggest that Snail could be used clinically as a marker for the tumor possessing potential for lymph node metastasis. Arecoline was capable of stimulating Snail expression in HOKs and OECM-1 cells. This suggests that areca quid chewing may contribute the pathogenesis of OSCCs via Snail expression. To examine the direct role of Snail in areca quid chewing-associated oral carcinogenesis, the specific inhibition of Snail by siRNA in arecoline-stimulated HOKs and OECM-1 will be determined in the further works. Moreover, this study represents that arecoline was capable of stimulating intracellular ROS generation and augmenting Snail expression in HOKs and OECM-1. This suggests that arecoline-induced Snail expression in HOKs and OECM-1 may be partly mediated by ROS. Arecoline may contribute the pathogenesis of OSCCs *via* Snail expression. In addition, NAC, curcumin, and EGCG significantly reduced the expression of Snail. These suggest that NAC, curcumin, and EGCG may play the antioxidative and cell protective role in the reduction of arecoline-induced Snail expression (Fig. 5). Therefore, studying the possiable mechanism involved in Snail expression may prove versatile. However, more detailed studies should be undertaken to clarify the agents that can regulate Snail in vivo and ex vivo.
