# Correction: Elevated Snail Expression Mediates Tumor Progression in Areca Quid Chewing-Associated Oral Squamous Cell Carcinoma *via* Reactive Oxygen Species

**DOI:** 10.1371/annotation/37a1c77e-e425-4f4b-932c-1e4163d815cd

**Published:** 2014-01-17

**Authors:** Shiuan-Shinn Lee, Chung-Hung Tsai, Cheng-Chia Yu, Yu-Chao Chang

Figure 2A was incorrectly posted as a duplicate of Figure 2C. Please view the corrected Figure 2 here: http://plosone.org/corrections/pone.0067985.g002.cn.tif

